# The AHA Recommendations for a Healthy Diet and Ultra-Processed Foods: Building a New Diet Quality Index

**DOI:** 10.3389/fnut.2022.804121

**Published:** 2022-04-11

**Authors:** Leandro Teixeira Cacau, Aline Marcadenti, Angela Cristine Bersch-Ferreira, Bernardete Weber, Jussara Carnevale de Almeida, Cíntia Corte Real Rodrigues, Paulo Andrade Lotufo, Isabela Martins Bensenor, Dirce Maria Marchioni

**Affiliations:** ^1^Departamento de Nutrição, Faculdade de Saúde Pública, Universidade de São Paulo, São Paulo, Brazil; ^2^Instituto de Pesquisa, Hospital do Coração, São Paulo, Brazil; ^3^Programa de Pós-Graduação em Ciências da Saúde (Cardiologia), Intituto de Cardiologia/Fundação Universitária de Cardiologia do Rio Grande do Sul, Porto Alegre, Brazil; ^4^Departamento de Nutrição, Faculdade de Medicina, Universidade Federal do Rio Grande do Sul, Porto Alegre, Brazil; ^5^Serviço de Nutrição e Dietética, Hospital de Clínicas de Porto Alegre, Porto Alegre, Brazil; ^6^Centro de Pesquisa Clínica e Epidemiológica, Hospital Universitário, Universidade de São Paulo, São Paulo, Brazil

**Keywords:** diet quality, diet index, cardiovascular health, ultra-processed foods, validation, nutritional epidemiology

## Abstract

The American Heart Association (AHA) has developed the concept of “ideal cardiovascular health” (ICH), a seven-component score, which includes health dietary metrics. Higher ultra-processed foods intake is related with several cardiometabolic and cardiovascular diseases. We propose to develop and validate the Cardiovascular Health Diet Index (CHDI), a diet quality index that combines the AHA's recommendations of a healthy diet for cardiovascular health and ultra-processed foods. We used dietary data obtained through a 114-item FFQ from 14,779 participants of the Brazilian Longitudinal Study of Adults Health (ELSA-Brasil). The CHDI had 11 components and a total score ranging from 0 to 110 points. Validation and reliability analyses were performed, including principal component analyses, association with selected nutrients, means differences between groups (for example, smokers vs. non-smokers), Cronbach's alpha, and linear regression analyses between CHDI and overall dietary quality. The mean CHDI was 57.1 points (95% CI 47.9:66.0). The CHDI had four dimensions; in addition, it was associated with nutrients related to cardiovascular health, and the points were significantly (*p* < 0.001) lower in smokers (52.1) than in non-smokers (57.8). Cronbach's alpha value was 0.50. After age and sex adjustment, the CHDI score remained associated with a higher overall dietary quality (β 0.87, 95%CI 0.84:0.89, *p* < 0.001). The CHDI proved to be valid and reliable for use, in addition to being associated with higher overall dietary quality. The use of CHDI is expected to assess the population's compliance with dietary recommendations for promoting cardiovascular health and preventing cardiovascular disease.

## Introduction

In 2010, the American Heart Association (AHA) proposed the “Strategic Impact Goals” with the aim of decreasing cardiovascular disease (CVD), mortality rates and improving cardiovascular health (CVH) in the United States by 2020 (1). In order to achieve these goals, and assess and monitor the population's cardiovascular health, the AHA developed the concept of “ideal cardiovascular health” (ICH), a seven-metric score, that includes four lifestyle factors: body mass index (BMI), smoking, physical activity, and the healthy diet score (HDS); and three health factors: blood pressure, fasting blood glucose, and total cholesterol (1).

Each year, the AHA issues an updated document with the latest statistics on CVD, its risk factors, and strategies to achieve a CVH status. Regarding diet, which is a key strategy for achieving CVH, it has proposed the HDS, which is a healthy dietary pattern characterized by five primary metrics (fruits and vegetables, fish, sodium, sweet sugar beverages, and whole grains) and three secondary metrics (nuts, seeds, and vegetables, processed meat, and saturated fat) (2). The HDS has a binary score (0 or 1 point) but can be scored alternatively (0 to 10 points). Individuals' HDS scores are classified as poor, intermediate, or ideal (2).

In the National Health and Nutrition Examination Survey (NHANES) diet assessment, the dietary quality assessed by HDS improved between 2003/2004 and 2015/2016, with a reduction from 56% to 48% in the prevalence of poor diets (2). Although this score was used to assess dietary quality in the NHANES study, only one study evaluated the effectiveness of this score taking into account healthy outcomes, showing that the compliance with the HDS recommendations may reduce the 20-year risk of mortality (3).

In addition, some initiatives comparing dietary quality indexes with CVD have already been described in the literature, such as the Healthy Eating Index-2015 (HEI-2015), and the alternate Mediterranean diet (aMed) score (4–6). Recently, ultra-processed food (UPFs) classification has also been used as an indicator of diet quality (7) and some studies demonstrated an association between higher UPF intake and poorer CVH (8–12). As a definition, UPFs are industrial formulations made entirely or predominantly of substances extracted from foods or laboratory-synthesized ingredients based on organic materials (13). The most used method to classify UPFs is the NOVA system, which classifies the foods based on the extent and purpose of the industrial processing they are subjected to (13). Although some diet indexes and UPFs are used to assess diet quality and its relationship with CVD, a diet index that combines these metrics is lacking and to the best of our knowledge, there is no AHA recommendation-based diet index in literature, which is adapted to local foods and different cultures, including foods related to CVD, such as UPFs. Thus, we aimed to describe the development and validation of an index based on the AHA Dietary Targets and HDS for defining CVH (2), considering the Brazilian food culture and including UPFs as a component related to CVD.

## Materials and Methods

### Study Design

This study is a cross-sectional analysis of the baseline data from Brazilian Longitudinal Study of Adult Health (ELSA-Brasil), which is a multicenter cohort of 15,105 civil men and women, aged between 35 and 74 years, who were active and retired workers from six institutions in six different Brazilian cities (São Paulo, Rio de Janeiro, Belo Horizonte, Vitória, Porto Alegre, and Salvador), from three major Brazilian regions (Northeast, Southeast, and South). ELSA-Brasil aimed to investigate the incidence and risk factors of CVD and diabetes. Baseline data from ELSA-Brasil were collected between August 2008 and December 2010. Details of the sample and data collection methods used in this study have been published previously (14, 15).

ELSA-Brasil was approved by the research ethics committees of all research centers. All participants volunteered and signed an informed consent form. This study was also approved by the research ethics committee of the School of Public Health of the University of São Paulo (number 3.970.703).

### Diet Assessment

Food consumption was assessed using a previously developed and validated semi-quantitative food frequency questionnaire (FFQ), with 114 food items (16, 17). This FFQ comprises the past 12 months, and the questions are structured into three sections: (1) food products/food preparations, (2) consumed products measurements, and (3) consumption frequencies with eight response options (more than 3 times/day, 2–3 times/day, once a day, 5–6 times a week, 2–4 times a week, once a week, 1–3 times a month, and never or almost never).

The daily consumption of each FFQ item (in g/day) was obtained by multiplying the portion size by the corresponding frequency. Food measurements were then converted into nutrient intakes using the United States Department of Agriculture (USDA) Food Composition Database, except when its values were outside the range of 80 to 120% from those described in the Brazilian Table of Food Composition, in these items the latter reference was used. For the present analysis, we disregarded participants missing food consumption information (*n* = 24) and those who were below the 1st and above the 99th percentile of dietary energy intake (*n* = 302), in order to exclude possibly invalid food intake data. The final sample comprised 14,779 individuals.

### Cardiovascular Health Diet Index

The Cardiovascular Health Diet Index (CHDI) was based on the HDS-AHA recommendations (2), with some adaptations to suit the Brazilian food culture, and inclusion of the components based on scientific evidence regarding protection (dairy products) or risk (red meat and UPFs) of CVD and other outcomes, such as type-2 diabetes mellitus (T2DM) (8–12, 18, 19). Briefly, the HDS-AHA (2) were divided into primary and secondary metrics. The primary metrics were composed of fruits and vegetables, fish and seafood, sodium, sweet sugar beverages (SSBs), and whole grains, while the secondary metrics were composed of nuts and legumes, processed meat, and saturated fat. However, in the CHDI, the following groups were considered: fruits, vegetables, fish and seafood, SSBs, whole grains, nuts, legumes, and processed meat, in addition to the inclusion of red meat, dairy products, and UPFs consumption. Sodium and saturated fat were not taken into account as the nutritional recommendations for reducing cardiovascular risk (such as T2DM) are now focused on dietary patterns and not on a particular nutrient. Supplementary Table 1 shows examples of foods included in the CHDI components and Supplementary Table 2 presents the portions and their associated values in grams.

The fruits, vegetables, fish, whole grains, legumes, nuts, and dairy groups were given scores from 0 to 10, where a score of 10 denoted consumption equal to or greater than recommended. Red meat, SSBs, processed meat, and UPF were scored inversely; a score of 0 denoted consumption equal to or above the recommended value. The final score had 11 items that ranged from 0 to 110 points (Table 1). The description of each components is as follows.

**Table 1:** Cardiovascular Health Diet Index components and standards for scoring.

**Component**	Standard for a maximum score of 10 points^*^	**Standard for a minimum score of 0 points**
Fruits	≥340 g/d	No fruits
Vegetables	≥180 g/d	No vegetables
Fish and seafood	≥28.6 g/d	No fish and seafood
Red meat	≤28.6 g/d	>28.6 g/d
SSBs	≤142.9 ml/d	>142.9 ml/d
Whole grains	≥90 g/d	No whole grains
Legumes	≥80g/d	No legumes
Nuts	≥12.9 g/d	No nuts
Processed meat	≤12.9 g/d	>12.9 g/d
Dairy	≥250 g/d	No dairy
UPF	≤4 points	≥23 points

* *For intermediate values were given a score proportional to the amount consumed. UPF, ultra-processed food*.

• Fruits: According to the HDS-AHA (2), the recommendation for fruits and vegetables is at least 4.5 cups per day. However, we decided to separate the recommendation of fruits and vegetables into two cups of fruit per day and 2.5 cups of vegetables per day. According to the USDA database, one cup of fruit is equivalent to 170 g, thus totaling 340 g per day for two cups. All fruits were included; however, we did not consider fruit juice as part of this group.

• Vegetables: As described above, the recommendation of 4.5 cups of fruits and vegetables was divided, and 2.5 cups of vegetables were recommended per day. According to the HDS-AHA (2), the vegetable group includes all vegetables, including tubers. Tubers were not included in the CHDI because they were not associated with a reduction in CVD. Therefore, the recommended value for the vegetable group was lowered, because we did not consider tubers. According to the USDA, a cup of vegetables is equal to 115 g, so we stipulated a recommendation of 180 g per day (equivalent to 1.5 cups/day). All vegetables were included, except tubers.

• Fish: According to the HDS-AHA (2), consumption of 200 g per week of fish is recommended. To standardize the scoring recommendations, weekly consumption was divided into daily consumption, resulting in a recommendation of 28.6 g per day. Fried, canned and boiled fish were included in this group.

• Red meat: Although the HDS-AHA (2) does not consider red meat in its recommendations, we have included it in the CHDI as there are associations between the consumption of red meat and the risk of CVD (18–20). The average recommended consumption of red meat described in the literature is 200 g per week. Thus, we adopted the cutoff point for the maximum daily consumption of 28.6 g. Beef and pork were included in this group.

• SSBs: According to the HDS-AHA metrics (2), the consumption of SSBs should be at most 1 L per week. In order to standardize the score recommendations, we changed the recommendation to daily, reaching a value of 142.9 mL per day. Soft drinks, sweetened natural juices, sweetened industrialized juices, and sweetened coffee were included in this group.

• Whole grains: According to the HDS-AHA metrics (2), the recommended consumption of whole grains is equivalent to 90 g. We have maintained this recommendation. Oats, brown bread, and brown rice were included in this group.

• Legumes: The HDS-AHA (2) recommends the consumption of ≥4 servings/week of nuts, seeds and legumes. For vegetables, 0.5 cups. According to the USDA, 0.5 cups of legumes are equal to 80 g. We therefore decided to establish a daily recommendation of 80 g of legumes. This group includes beans, chickpea, and lentils.

• Nuts: For nuts and seeds, the HDS-AHA metrics (2) recommended 90 g, per week. To standardize the CHDI metrics, we defined the recommendation as daily one, 12.9 g.

• Processed meat: According to the HDS-AHA (2), the recommendation for processed meat consumption should be a maximum of 100 g per week. To standardize the recommendations, we recommended a maximum of 12.9 g per day.

• Dairy: Although the HDS-AHA (2) does not consider dairy in its recommendations, owing to the inconclusive results regarding the effects of these foods on CVD, we have included it in the CHDI due to the evidence of the inverse association between the consumption of dairy products and diabetes mellitus, an important risk factor for CVD. Therefore, we established a cutoff point of 250 g of dairy products per day. All types of milk, cheese, and yogurt were included in this group.

• UPFs: The consumption of UPFs was included in the score because of the evidence of an association between high consumption of these foods and CVD (8–12, 21). The NOVA score was used for UPFs consumption. Briefly, this score was proposed to assess the UPF consumption by the participants of an ongoing cohort study in Brazil: the NutriNet-Brasil study (22). The NOVA score is calculated from the sum of the reported UPFs and can vary from 0 to 23 points (22). We adopted the cutoff point of 4, as the NOVA score shows that when the NOVA score is >5, there is a linear association with the percentage of contribution from UPF (~>50%) on the daily total energy intake (22). UPFs were classified according to the NOVA classification (13).

### Validity and Reliability of the CHDI

The performance of the CHDI was measured using strategies to assess construct validity and reliability, as proposed by Reedy et al. (23). In addition, we checked the validity of the CHDI by relating it to the overall dietary quality evaluated using a national tool (24).

#### Construct Validity

In order to assess the construct validity, we used linear regression models adjusted for sex and age to investigate the correlation of the score with nutrients related to cardiovascular health, such as total fat, saturated fat, cholesterol, monounsaturated fat (MUFA), polyunsaturated fat (PUFA), carbohydrate, total protein, animal and vegetable sources of protein, fiber, added sugar, sodium and total energy intake. We also used Pearson's correlation between total score and components with total energy to assess whether the score evaluates the compliance with AHA metrics regardless of diet quantity, and principal component analysis (PCA) to verify whether the structure of the score has another factor that explained the data variability. In the PCA, the matrix was obtained using varimax rotation, eigenvalues >1, and a scree plot shape was used to determine the number of factors (25).

#### Concurrent-Criterion Validity

Concurrent criterion validity was also assessed by comparing the average score among the groups with known differences in diet quality, such as smokers and non-smokers, adults and the elderly, men and women of physically active individuals and individuals with sedentary lifestyle. For discrimination analysis, comparisons among groups were performed using a *t*-test or analysis of variance (ANOVA). Internal reliability was assessed using Cronbach's alpha coefficients and Pearson's correlations between components.

#### Internal Reliability

Cronbach's alpha was used to assess internal reliability. This statistic assesses the average of the correlations among all possible combinations, in this case, the 11 CHDI components. We also performed item-item correlations between the components to better understand their relationships.

#### Overall Dietary Quality

Finally, we assessed the relationship between the CHDI and the overall dietary quality, using a national tool—the Brazilian Healthy Eating Index-Revised (BHEI-R) (24, 26). This index is composed of 12 components: nine food groups (total fruit, whole fruit, total vegetables, dark green and orange vegetables, total grains, whole grains, milk and dairy, meat, eggs and legumes, and oils), two nutrients (saturated fat and sodium), and the sum of energy from solid fat, alcohol, and added sugar (the SoFAAS component). The BHEI-R can range from 0 to 100 and is estimated per 1,000 kcal. The relationship between the CHDI and BHEI-R was explored using linear regression models adjusted for sex and age.

### Statistical Analysis

All analyses were performed using Stata Statistical Software (release 14, 2015, StataCorp LP, College Station, Texas, USA), and the level of significance was set at *p* < 0.05.

## Results

The mean total CHDI score was 57.1 points (95% confidence interval [95% CI] 56.9:57.3), on a scale that ranged from 0 to 110 points, and had a normal distribution (Supplementary Figure 1). Some components had higher average scores, such as fruits, vegetables, fish and seafood, and legumes, whereas the red meat, whole cereals, SSBs, and nut components had lower average scores (Supplementary Table 3).

The total CHDI score was, in an expected way, associated with nutrients related to cardiovascular health, showing a positive association (*p* < 0.001) with carbohydrate, protein, vegetable protein source, PUFA, and fiber, and a negative association (*p* < 0.001) with total fat, MUFA, saturated fat, cholesterol, added sugar, and sodium (Table 2). The correlations between each CHDI component and the total energy intake were all low. The highest absolute correlation was between energy and processed meat (−0.32), which was expected (Supplementary Table 4). The correlation between total energy and total score was also low (−0.13).

**Table 2:** Association between Cardiovascular Health Diet Index score and selected nutrients. ELSA-Brasil, 2008–2010.

**Nutrient**	**β^a^**		**95% CI**	***p*-value**
Energy (kcal)	0.0018	0.0015	0.0021	<0.001
Carbohydrate (% kcal)	0.2588	0.2299	0.2876	<0.001
Protein (% kcal)	0.7309	0.6524	0.8095	<0.001
Animal protein source (% kcal)	0.0302	−0.0360	0.0965	0.371
Vegetable protein source (% kcal)	1.6230	1.5043	1.7417	<0.001
PUFA (% kcal)	0.2449	0.1021	0.3876	<0.001
Fiber g/1,000 kcal	0.9881	0.9486	1.0276	<0.001
Total fat (% kcal)	−0.3107	−0.3525	−0.2689	<0.001
MUFA (% kcal)	−0.8536	−0.9611	−0.7462	<0.001
Saturated (% kcal)	−0.7033	−0.7900	−0.6166	<0.001
Cholesterol mg/1,000 kcal	−0.0554	−0.0612	−0.0497	<0.001
Added sugar g/1,000 kcal	−0.3261	−0.3453	−0.3068	<0.001
Sodium mg/1,000 kcal	−0.0023	−0.0027	−0.0018	<0.001

a *Linear regression adjusted by sex and age*.

The PCA revealed several factors that explained the CHDI variability. The scree plot illustrated that no single linear combination of the 11 CHDI components was responsible for a significant proportion of the covariance of the data. Supplementary Figure 2 shows the presence of four factors with eigenvalues >1, and that the line seems to stagnate after the fifth factor. Higher averages of CHDI were observed among women, the elderly, non-smokers, and those with a moderate and vigorous level of physical activity when compared to men, adults, smokers, and those with a low level of physical activity, respectively (Table 3).

**Table 3:** Characteristics of the individuals included in the study and their Cardiovascular Health Diet Index scores. ELSA-Brasil, 2008–2010.

	**Individuals**		**CHDI**		
**Characteristics**	**n**	**%**	**mean**	**95% CI**	*p*-value^*^
Sex					<0.001
Men	6,724	45.5	55.3	54.9–55.6	
Women	8,056	54.5	58.6	58.3–58.9	
Age group					<0.001
Adults	11,598	78.5	55.8	55.5–56.0	
Elderly	3,182	21.5	62.0	61.5–62.4	
Smoking status					<0.001
Non-smokers	12,863	87.0	57.8	57.6–58.1	
Current smokers	1,916	13.0	52.1	51.6–52.3	
Physical activity level^§^					<0.001
Low	11,220	77.0	55.8	55.6–56.0	
Moderate	2,029	14.0	61.2	60.6–61.8	
Vigorous	1,314	9.0	61.8	61.1–62.6	

* *t-test or ANOVA*.

§ *n = 14,563*.

Cronbach's alpha was 0.50, and the item-item correlations showed that the higher correlations were found between the components processed meat and UPF (0.40), fruits and vegetables (0.35), and processed meat and red meat (0.28), which was expected. Regardless of the expected correlations, all the other correlations were low (Supplementary Table 4). Finally, the CHDI score was significantly associated with the overall dietary quality, as assessed using the BHEI-R. The mean BHEI-R was 70.3 points (95% CI 70.2:70.5). After adjusting for age and sex, the CHDI score remained positively associated with overall dietary quality (Table 4).

**Table 4:** Association between Cardiovascular Health Diet Index and overall dietary quality. ELSA-Brasil, 2008–2010.

**Regression models**	**Bivariate**			
	**β**	**95% CI**		***p*-value**
**Model**				
BHEI-R	0.89	0.87	0.92	<0.001
BHEI-R^*^	0.87	0.84	0.89	<0.001

* *Model adjusted for age and sex. BHEI-R, Brazilian Healthy Eating Index Revised*.

## Discussion

In this study, we developed and validated an index to assess adherence to a healthy diet for cardiovascular health based on the AHA recommendations, with some adaptations to Brazilian food culture, including foods associated with CVD, and its risk factors, such as UPFs and red meat. We found that the CHDI performed satisfactorily in terms of validity and reliability and was associated with higher overall dietary quality. To date, this is the first dietary assessment index that combines healthy eating recommendations for cardiovascular health and UPF in its metrics. CHDI is in line with what the AHA proposes in its statement launched in 2021 “2021 Dietary Guidance to Improve CVH,” where for the first time, the recommendation to reduce the consumption of UPFs aligns with previous recommendations for a healthy diet for cardiovascular health (27).

The CHDI has been associated with nutrients related to cardiovascular health in the literature, such as being positively associated with carbohydrates, total protein, vegetable protein source, PUFA and fiber intake, while it has been negatively associated with total fat, MUFA, saturated fat, cholesterol, added sugars, and sodium. Similar results were found with other dietary quality indices previously described in the literature (28, 29), and with an index developed based on the Brazilian Cardioprotective Nutritional Program (BALANCE) recommendations, an educational intervention aimed at improving the consumption of foods available in Brazil with potential cardioprotective function (30, 31). Therefore, the BALANCE diet index (BALANCE DI) (32) was proposed to assess the population's compliance with the program's recommendations. In its validation process, higher BALANCE DI scores were inversely associated with energy, total fat, MUFA, and cholesterol intake and positively associated with carbohydrate and fiber intake (32). Results similar to those were found among CHDI scores with these nutrients. The CHDI showed a positive association with energy intake, however, this association was extremely low. No association was found with the animal protein sources.

Construct validity was confirmed according to criteria established in the literature (23). The PCA presented four components and showed no evidence of a linear combination responsible for explaining a substantial part of the index variation. The correlations between the various component scores varied from low to moderate. In addition, construct validity was supported by the CHDI being able to distinguish individuals with known disparities in diet quality; women, the elderly, non-smokers, and those who practice physical activity had higher scores than men, adults, smokers, and sedentary people. These results demonstrated good construct validity in the CHDI and are similar to the results found in the construct validity analyses of other indices described in the literature (23, 26, 33, 34).

In addition, the CHDI presented a reliability coefficient of 0.50, assessed by internal consistency (Cronbach's alpha). This statistic measures the internal consistency of an index, and values > 0.70 indicate accepted reliability (33). Nevertheless, some studies with diet quality indices found alpha values ranging from 0.22 to 0.68 (33–38). However, it is important to consider that the sample and index characteristics can affect the results of Cronbach's alpha. For example, if the index is uni- or multidimensional or if the sample is heterogeneous (33). As the CHDI proved to be a multidimensional index, in addition to the heterogeneous population in our study, we can consider our result acceptable, according to the aforementioned characteristics. Therefore, this result is consistent, as diet is a complex and multidimensional construct (33).

The total CHDI score was positively associated with the overall dietary quality assessed using a previously validated index in Brazil, the BHEI-R (24, 26). This result demonstrates that CHDI can capture the diet quality of the individuals and that those with better diet quality present higher adherence to a diet that promotes CVH. This result is interesting, as the studies in the literature demonstrate an association between higher diet quality and cardiovascular diseases (2). This result suggests that the CHDI may also present positive results with CVH outcomes (39).

Diet quality indices can be used to monitor the compliance with dietary guidelines and the overall dietary quality of a population (40, 41). In addition, they can be used to make comparisons within and between different populations and to test whether the evaluated dietary recommendations have a protective effect on health (40, 41). Despite this, the development of the dietary quality index can be challenging because it involves many arbitrary choices, such as its construction, its components, cutoff points, and the score scale used (39–41).

Nevertheless, the CHDI has strengths. The scoring criterion used is a gradual score, which allows a better distinction between the individual's scoring degrees, favoring interpersonal distribution (41). Moreover, it allows for a more refined association between the diet quality and health outcomes. The cutoff points followed the AHA recommendations and were based on scientific evidence. Another strength is the inclusion of UPFs metric, in which, although there is no quantification of consumption and an established cutoff point, presents the frequency of consumption and has been recognized by the World Health Organization for its potential integrating use as an instrument for evaluating diet quality (42). The CHDI presented good validity and reliability in results, in addition to being associated with higher overall dietary quality. We used food consumption data obtained from a previously validated FFQ that evaluated the usual consumption over the preceding 12 months. Finally, the CHDI was developed in a multicenter study with a large ethnically and socially diverse population, similar to that of heterogeneous populations, mainly of middle income living in large Brazilian cities.

However, some limitations should be acknowledged. The food consumption data obtained from the FFQ have some biases, such as the finitude of the list of foods and the self-report method, even though it has been previously validated and is one of the most used instruments in epidemiological studies to assess the relationship between diet and health outcomes. Although the validity and reliability criteria presented were satisfactory, the CHDI was not evaluated for its predictive validity, such as its ability to predict death and/or illness. However, these analyses are planned and will be carried out soon, as ELSA-Brasil is an ongoing cohort study.

In conclusion, we developed and validated an index based on AHA recommendations and included components based on scientific evidence of its association with cardiovascular risk outcomes, such as red meat and UPFs. The CHDI was evaluated for construct validity, concurrent validity, and reliability, and presented satisfactory results, in addition to being associated with overall dietary quality. The use of CHDI is expected to assess a population's compliance with dietary recommendations to promote CVH and preventing CVD. Additional study of the index should be performed to assess predictive validity. In addition, it would be interesting to assess whether the CHDI performs well in clinical trials and whether it may reflect changes in CVH over time.

## Data Availability Statement

The analytic code of the CHDI computation will be made available upon request pending.

## Ethics Statement

The studies involving human participants were reviewed and approved by Research Ethics Committee of the School of Public Health of the University of São Paulo (number 3.970.703). The patients/participants provided their written informed consent to participate in this study.

## Author Contributions

LC, AM, AB-F, and DM: study concept, design, and interpretation of the data. LC: data analysis and drafting of the manuscript. AM, AB-F, BW, JA, CR, IB, PL, and DM: critical revision of the manuscript for important intellectual content. All authors read and approved the final version of this manuscript.

## Funding

The ELSA-Brasil baseline study was supported by the Brazilian Ministry of Health (Science and Technology Department), the Brazilian Ministry of Science and Technology, and National Research Council grants 01 06 0010.00 RS, 01 06 0212.00 BA, 01 06 0300.00 ES, 01 06 0278.00 MG, 01 06 0115.00 SP, and 01 06 0071.00 RJ). The research center of São Paulo was also supported by São Paulo Research Foundation (FAPESP; grant 2011/12256-4). LC receive a scholarship from the FAPESP (grant number 2019/13424-0). The Public Health Nutrition Graduate Program is supported by the Coordination of Superior Level Staff Improvement (CAPES). HCor/PROADI-SUS, which funds the NUGLIC and NUPRESS studies and the CAPES supported the English language revision of this manuscript. No funding agencies had a role in the study design, data collection, analysis, decision to publish, or preparation of the article.

## Conflict of Interest

The authors declare that the research was conducted in the absence of any commercial or financial relationships that could be construed as a potential conflict of interest.

## Publisher's Note

All claims expressed in this article are solely those of the authors and do not necessarily represent those of their affiliated organizations, or those of the publisher, the editors and the reviewers. Any product that may be evaluated in this article, or claim that may be made by its manufacturer, is not guaranteed or endorsed by the publisher.
