# Supplementary material for: The AHA Recommendations for a Healthy Diet and Ultra-Processed Foods: Building a New Diet Quality Index
Source: Front Nutr. 2022 Apr 11;9:804121. doi: 10.3389/fnut.2022.804121 (PMC9036106; doi:10.3389/fnut.2022.804121)

Supplementary Material

| **Table S1.** Example of foods and ingredients included in the CHDI components. | |
| --- | --- |
| Components | Example of included items |
| Fruits | All fruits, except fruits juices |
| Vegetables | All vegetables, except tubers. |
| Fish and seafood | Boiled and fried fish |
| Red meat | Beef and pork |
| SSB | Sweetened juices, sweetened coffees and teas, sodas. |
| Whole cereals | Whole bread, brown rice and oatmeal |
| Legumes | Beans and lentils |
| Nuts | Nuts and peanuts |
| Processed meat | Processed meat, such as sausage and ham |
| Dairy | All dairy, such as milks, yogurts and cheeses. |
| Ultraprocessed food | All ultra-processed foods classified by NOVA (e.g., sweet bread, salty and sweet biscuits, margarine etc.) |

| **Table S2**. Number of portions of the Cardiovascular Health Diet Index components, considering the maximum score. | | |
| --- | --- | --- |
| Component | Standard for a maximum score | Number of portions |
| Fruits | >340g/d | ≥ 2 portions of 170g/day |
| Vegetables | >180g/d | ≥ 6 portions of 30g/day |
| Fish and seafood | >28.6g/d | ≥ 2 portions of 100g/week |
| Red meat | <28.6g/d | < 2 portions of 100g/week |
| SSB | <142.9ml/d | < 1 liter/week |
| Whole cereals | >90g/d | ≥ 3 portions of 30g/day |
| Legumes | >80g/d | ≥ 1 portion of 80g/day |
| Nuts | >12.9g/d | ≥ 3 portions of 30g/week |
| Processed meat | <12.9g/d | ≤ 3 portions of 30g/week |
| Dairy | >250g/d | ≥ 1 portion of 250g/day |
| Ultraprocessed food | <4 points | < 4 units/day |
|  | | |

**Figure S1.** Normal distribution of the Cardiovascular Health Diet Index. ELSA-Brasil, 2008–2010.

**
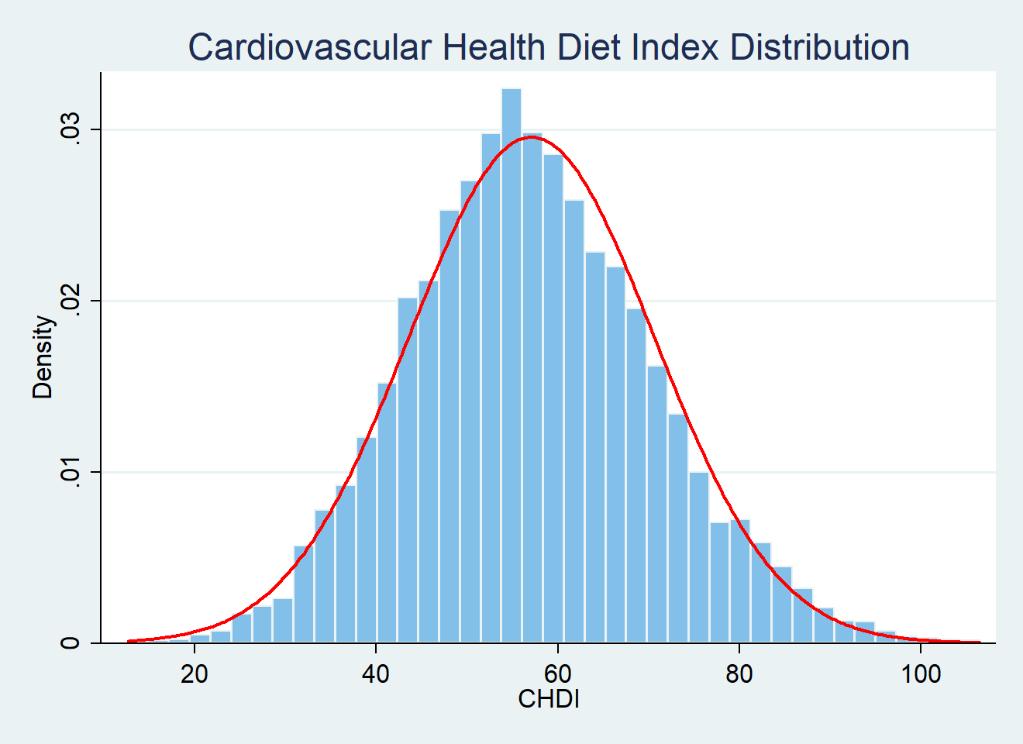
**

| **Table S3.** Descriptive analysis of Cardiovascular Health Diet Index components (values expressed as mean and standard deviation, and median and interquartile range). ELSA-Brasil, 2008-2010. | | | | | |
| --- | --- | --- | --- | --- | --- |
| Components | *Maximum points* | Mean | SD | Median | IQR |
| Fruits | *10* | 7.7 | 2.9 | 9.8 | 5.6 – 10 |
| Vegetables | *10* | 7.3 | 2.8 | 8.0 | 5.1 – 10 |
| Fish and seafood | *10* | 7.6 | 3.7 | 10 | 4.9 – 10 |
| Red meat | *10* | 1.2 | 2.7 | 0 | 0 – 0 |
| SSBs | *10* | 1.4 | 2.9 | 0 | 0 – 0.7 |
| Whole grains | *10* | 2.8 | 3.5 | 1.0 | 0 – 5 |
| Legumes | *10* | 7.2 | 3.8 | 10 | 4.4 – 10 |
| Nuts | *10* | 2.5 | 3.4 | 1.0 | 0 – 3.3 |
| Processed meat | *10* | 4.8 | 4.0 | 5.1 | 0 – 9.2 |
| Dairy | *10* | 6.9 | 3.7 | 9.6 | 3.2 – 10 |
| UPF | *10* | 6.6 | 1.6 | 6.5 | 5.7 – 7.4 |
| Total score | *0 - 110* | 57.1 | 13.5 | 56.5 | 47.9 – 66.0 |
| SD: standard deviation. IQR: interquartile range. UPF: ultra-processed food. | | | | | |

**Table S4**. Correlation between Cardiovascular Health Diet Index score components and each component with total energy intake. ELSA-Brasil, 2008-2010.

| Component | Fruits | Vegetables | Fish | Red meat | SSB | Whole cereals | Legumes | Nuts | Processed meat | Dairy | UPF |
| --- | --- | --- | --- | --- | --- | --- | --- | --- | --- | --- | --- |
| Vegetables | 0.35** |  |  |  |  |  |  |  |  |  |  |
| Fish | 0.16** | 0.18** |  |  |  |  |  |  |  |  |  |
| Red meat | 0.03* | -0.01 | -0.05** |  |  |  |  |  |  |  |  |
| SSB | 0.08** | 0.09** | 0.03* | 0.15** |  |  |  |  |  |  |  |
| Whole cereals | 0.16** | 0.16** | 0.10** | 0.16** | 0.18** |  |  |  |  |  |  |
| Legumes | 0.00 | 0.05** | 0.00 | -0.10** | -0.09* | -0.07** |  |  |  |  |  |
| Nuts | 0.13** | 0.12** | 0.10** | 0.07** | 0.14** | 0.23** | -0.08** |  |  |  |  |
| Processed meat | 0.09** | 0.02* | -0.05** | 0.28** | 0.14** | 0.17** | -0.11** | 0.04** |  |  |  |
| Dairy | 0.13** | -0.08** | 0.03* | -0.02* | 0.01 | 0.10** | 0.03** | 0.03* | 0.02* |  |  |
| UPF | 0.00 | 0.02* | -0.08** | 0.14** | 0.16** | -0.00 | -0.05** | -0.09** | 0.40** | -0.06** |  |
| Energy | -0.04** | 0.19** | 0.16** | -0.22** | -0.20** | -0.06** | -0.01 | 0.04** | -0.32** | 0.14** | -0.23** |

*p<0.05 **p<0.001

**Figure S2.** Scree plot from principal components analysis (PCA) of Cardiovascular Health Diet Index. ELSA-Brasil, 2008-2010.


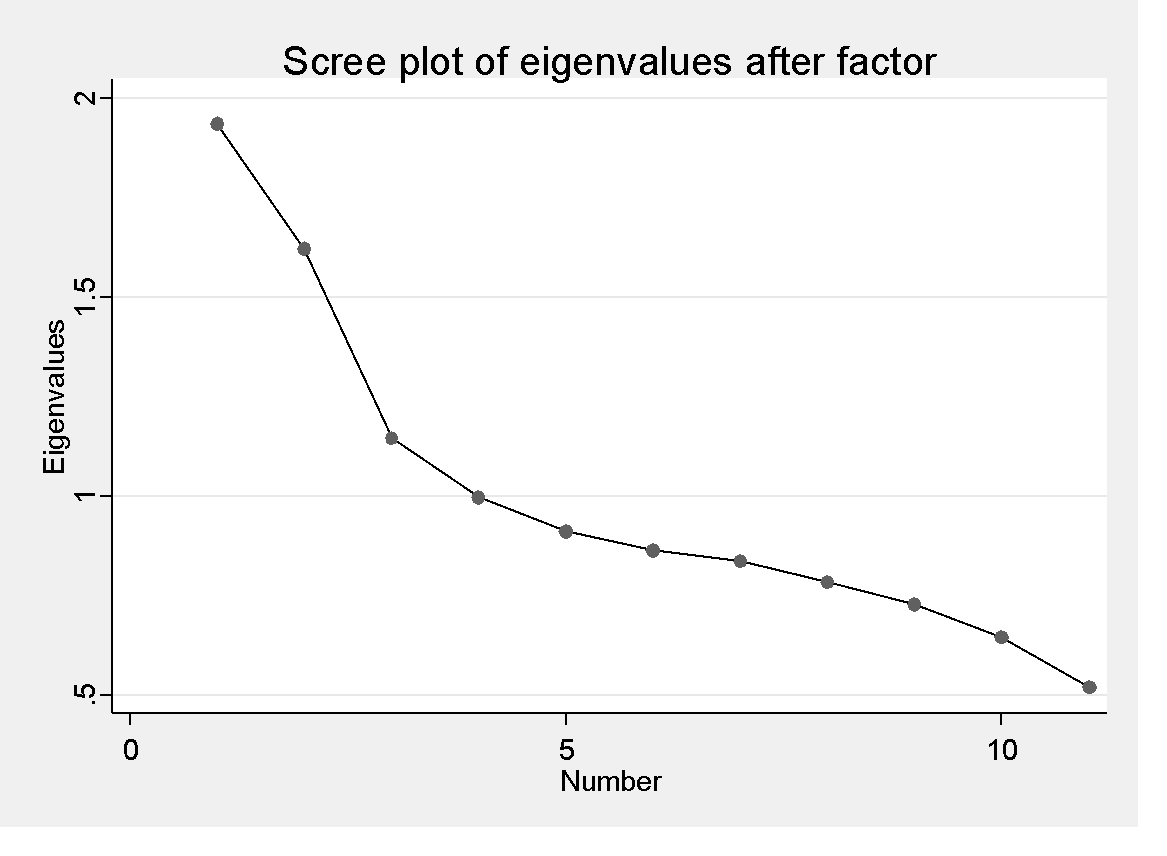

Supplement: Supplementary file 1 [file Data_Sheet_1.docx]
